# Supplementary material for: Modelling optimal vaccination strategy for SARS-CoV-2 in the UK
Source: PLoS Comput Biol. 2021 May 6;17(5):e1008849. doi: 10.1371/journal.pcbi.1008849 (PMC8101958; doi:10.1371/journal.pcbi.1008849)
Supplement: S1 Text — (PDF) [file pcbi.1008849.s001.pdf]

Supporting Information
Modelling optimal vaccination strategy for SARS-CoV-2 in the UK

[†]Sam Moore[*], [†]Edward M. Hill, [†]Louise Dyson, [†]Michael J. Tildesley, [†]Matt J. Keeling[*]

[†]The Zeeman Institute for Systems Biology & Infectious Disease Epidemiology Research, School of Life Sciences and Mathematics Institute, University of Warwick, Coventry, United Kingdom.

*Samuel.E.Moore@warwick.ac.uk SM, M.J.Keeling@warwick.ac.uk MJK

Table of Contents

1 The mathematical model **2**

2 Public Health Measurable Quantities **6**

3 Parameter Inference **7**

4 Modelling social distancing **8**

5 QALY losses **9**

1 The mathematical model

We used a compartmental age-structured model, developed to simulate the spread of SARS-CoV-2 within ten regions of the UK (seven regions in England: East of England, London, Midlands, North East & Yorkshire, North West, South East and South West; and the devolved nations: Northern Ireland, Scotland and Wales) [1], with parameters inferred to generate a good match to deaths, hospitalisations, hospital occupancy and serological testing [2]. The model population is stratified by age, with force of infection determined by the use of an age-dependent (who acquires infection from whom) social contact matrix for the UK [3, 4]. Additionally, we allow susceptibility and the probabilities of becoming symptomatic, being hospitalised and the risk of dying to be age dependent; these are matched to UK outbreak data. Finally, we account for the role of household isolation, by separating primary and secondary infections within a household (more details may be found in [1]). This allows us to capture household isolation by preventing secondary infections from playing a further role in onward transmission. Model parameters were inferred on a regional basis using regional time series of recorded daily hospitalisation numbers, hospital bed occupancy, ICU occupancy and daily deaths [2].

Model description

We first show the underlying system of equations that account for the transmission dynamics, including symptomatic and asymptomatic transmission, household saturation of transmission and household quarantining. The population is stratified into multiple compartments: individuals may be susceptible (S), exposed (E), infectious with symptoms (I), or infectious and either asymptomatic or with very mild symptoms (A). Asymptomatic infections are assumed to transmit infection at a reduced rate given by τ. To some extent, the separation into symptomatic (I) and asymptomatic (A) within the model is somewhat artificial as there are a wide spectrum of symptom severities that can be experienced.

We let superscripts denote the first infection in a household (F), a subsequent infection from a symptomatic household member (SI) and a subsequent infection from an asymptomatic household member (SA). A fraction (H) of the first detected cases (necessarily symptomatic) in a household are quarantined (QF), as are all their subsequent household infections (QS) - we ignore the impact of household quarantining on the susceptible population as the number in quarantine is assumed small compared with the rest of the population. The recovered class is not explicitly modelled, although it may become important once we have a better understanding of the duration of immunity. We omitted natural demography and disease-induced mortality in the formulation of the epidemiological dynamics. We then extended the model formulation to capture a range of vaccination scenarios.

The full equations are given by

$$
\begin{aligned}
\frac{dS_{a,v1}}{dt} &= -\left(\lambda^F_{a,v1} + \lambda^{SI}_{a,v1} + \lambda^{SA}_{a,v1} + \lambda^{Q}_{a,v1}\right)\frac{S_{a,v1}}{N_a}, \\
\frac{dE^F_{1,a}}{dt} &= \lambda^F_{a,v1}\frac{S_{a,v1}}{N_a} - M\varepsilon E^F_{1,a}, \\
\frac{dE^{SI}_{1,a}}{dt} &= \lambda^{SI}_{a,v1}\frac{S_{a,v1}}{N_a} - M\varepsilon E^{SI}_{1,a}, \\
\frac{dE^{SA}_{1,a}}{dt} &= \lambda^{SA}_{a,v1}\frac{S_{a,v1}}{N_a} - M\varepsilon E^{SA}_{1,a}, \\
\frac{dE^{Q}_{1,a}}{dt} &= \lambda^{Q}_{a,v1}\frac{S_{a,v1}}{N_a} - M\varepsilon E^{Q}_{1,a}, \\
\frac{dE^{X}_{m,a}}{dt} &= M\varepsilon E^{X}_{m-1,a} - M\varepsilon E_{m,a}, \qquad X \in \{F, SI, SA, Q\} \\
\frac{dI^F_a}{dt} &= d_{a,v2}(1-H)M\varepsilon E^F_{M,a} - \gamma I^F_a, \\
\frac{dI^{SD}_a}{dt} &= d_{a,v2}M\varepsilon E^{SI}_{M,a} - \gamma I^{SI}_a, \\
\frac{dI^{SU}_a}{dt} &= d_{a,v2}(1-H)M\varepsilon E^{SA}_{M,a} - \gamma I^{SA}_a, \\
\frac{dI^{QF}_a}{dt} &= d_{a,v2}HM\varepsilon E^{F}_{M,a} - \gamma I^{QF}_a, \\
\frac{dI^{QS}_a}{dt} &= d_{a,v2}HM\varepsilon E^{SA}_{M,a} + d_{a,v2}\varepsilon E^Q_a - \gamma I^{QS}_a, \\
\frac{dA^F_a}{dt} &= (1-d_{a,v2})M\varepsilon E^F_{M,a} - \gamma A^F_a, \\
\frac{dA^S_a}{dt} &= (1-d_{a,v2})M\varepsilon (E^{SI}_{M,a} + E^{SA}_{M,a}) - \gamma A^S_a, \\
\frac{dA^Q_a}{dt} &= (1-d_{a,v2})M\varepsilon E^Q_{M,a} - \gamma A^Q_a,
\end{aligned}
$$

Here we have included M latent classes, giving rise to an Erlang distribution for the latent period, while the infectious period was exponentially distributed. Throughout we have taken $M = 3$.

The forces of infection which govern the non-linear transmission of infection obey:

$$
\begin{aligned}
\lambda^F_{a,v1} &= \sigma_{a,v1}\sum_b \left(I^F_b + I^{SI}_b + I^{SA}_b + \tau(A^F_b + A^S_b)\right)\beta^N_{ba}, \\
\lambda^{SI}_{a,v1} &= \sigma_{a,v1}\sum_b I^F_b \beta^H_{ba}, \\
\lambda^{SA}_{a,v1} &= \sigma_{a,v1}\tau\sum_b A^F_a \beta^H_{ba}, \\
\lambda^{Q}_{a,v1} &= \sigma_{a,v1}\sum_b D^{QF}_b \beta^H_{ba},
\end{aligned}
$$

where β^H represents household transmission and $\beta^N = \beta^S + \beta^W + \beta^O$ represents all other transmission locations, comprising school-based transmission (β^S), work-place transmission (β^W) and transmission

in all other locations (β^O). These matrices are taken from Prem *et al.* [4] to allow easily translation to other geographic settings, although other sources such as POLYMOD [3] could be used.

Two key parameters, together with the transmission matrix, govern the age-structured dynamics: σ_a corresponds to the age-dependent susceptibility of individuals to infection; d_a the age-dependent probability of displaying symptoms (and hence potentially progressing to more severe disease). Both of these are also modified by the vaccine status, such that those that have received a dose of vaccine have a lower risk of infection and a lower risk of developing symptoms. The action of vaccine on the parameter σ captures the transmission blocking aspect of the vaccine, while the action on d captures the efficacy against disease. We also define τ as the reduced transmission from asymptomatic infections compared to symptomatic infections; given the probability of displaying symptoms is less in the younger age groups, this parameter shapes the role of younger ages in onward transmission.

We assume that all within household transmission originates from the first infected individual within the household (denoted with a superscript F, or QF if they become quarantined). This allows us to assume that secondary infections within a household in isolation (denoted with a superscript QS or Q) play no further role in any of the transmission dynamics. As a consequence, high levels of household isolation can drive the epidemic extinct, even if within household transmission is high – an effect not achievable with the standard SEIR-type modelling approach. This improved methodology also helps to capture to some degree household depletion of susceptibles (or saturation of infection), as secondary infections in the household are incapable of generating additional household infections.

Amendments to within-household transmission

We require our model to capture both individual level quarantining of infected individuals and isolation of households containing identified cases. In a standard ODE framework this level of household structure is only achievable at large computational expense [5, 6]. Thus, we instead made a relatively parsimonious approximation to achieve a comparable effect.

Given the novelty of the additional household structure that is included in this model, we clarify in more detail here the action of this formulation. We give a simpler set of equations (based on a standard SIR model) that contains a similar household structure; in particular, we take the standard SIR model and split the infected class into those first infected within a household (I_F) and subsequent infections (I_S):

$$\begin{aligned}
\frac{dS}{dt} &= -\beta^H S I_F - \beta^O S(I_F + I_S) \\
\frac{dI_F}{dt} &= \beta^O S(I_F + I_S) - \gamma I_F S \\
\frac{dI_S}{dt} &= \beta^H S I_F - \gamma I_S \\
\frac{dR}{dt} &= \gamma(I_F + I_S)
\end{aligned}$$

where the transmission rate is also split into within household transmission β^H and all other transmission β^O (i.e out-of-household transmission). Again, we make the assumption that only the first infection in any household generates infections within the household. We compare this to the SIR

model without this additional structure:

$$\frac{dS}{dt} = -\widehat{\beta}^H SI - \widehat{\beta}^O SI$$
$$\frac{dI}{dt} = \widehat{\beta}^H SI + \widehat{\beta}^O SI - \gamma I$$
$$\frac{dR}{dt} = \gamma I$$

where we retain the split in transmission type.

The early growth rate of the two models are $\widehat{r} = \widehat{\beta}^H + \widehat{\beta}^O - \gamma$ for the simple SIR model, and $r = \frac{1}{2}\left[\beta^O - 2\gamma + \sqrt{\beta^{O^2} + 4\beta^O\beta^H}\right]$ for the household structured version. From this simple comparison, it is clear that for the simple model the growth rate can remain positive even when control measures substantially reduce transmission outside the home ($\widehat{\beta}^O$ gets reduced), whereas in contrast for the structured version there is always a threshold level of transmission outside the household ($\beta^O_c = \gamma^2/(\beta^H + \gamma)$) that is needed to maintain positive growth.

For both the simple model given here and the full COVID-19 model, the inclusion of this additional household structure reduces the amount of within-household transmission compared to a model without this structure — as only the initial infection in each household (I_F) generates secondary within-household cases. It is therefore necessary to rescale the household transmission rate β^H to obtain the appropriate average within-household attack rate. For the full COVID-19 model, we find that a simple multiplicative scaling to the household transmission ($\beta^H \to z\beta^H$, $z \approx 1.3$) generates a comparable match between the new model and a model without this household structure – even when age structure is included.

Relationship between age-dependent susceptibility and detectability

We interlink age-dependent susceptibility, σ_a, and detectability, d_a, by a quantity Q_a. Q_a can be viewed as the scaling between force of infection and symptomatic infection. Taking a next-generation approach, the early dynamics would be specified by:

$$R_0 D_a = d_a \sigma_a \beta^N_{ba} \left(D_a + \tau U_a\right)/\gamma \qquad R_0 U_a = (1 - d_a)\sigma_a \beta^N_{ba} \left(D_a + \tau U_a\right)/\gamma$$

where D_a measures those with detectable infections, which mirrors the early recorded age distribution of symptomatic cases. Explicitly, we let $d_a = \frac{1}{\kappa} Q_a^{(1-\alpha)}$ and $\sigma_a = \frac{1}{k} Q_a^{\alpha}$. As a consequence, $Q_a = \kappa k d_a \sigma_a$; where the parameters κ and k are determined such that the oldest age groups have a 90% probability of being symptomatic ($d_{>90} = 0.90$) and such that the basic reproductive ratio from these calculations gives $R_0 = 2.7$.

2 Public Health Measurable Quantities

The main model equations focus on the epidemiological dynamics, allowing us to compute the number of symptomatic and asymptomatic infectious individuals over time. However, these quantities are not directly measured - and even the number of confirmed cases (the closest measure to symptomatic infections) is highly biased by the testing protocols at any given point in time. It is therefore necessary to convert infection estimates into quantities of interest that can be compared to data. We considered seven such quantities which we calculated from the number of new symptomatic infections on a given day I_a^d.

1. **Hospital Admissions:** An age-dependent fraction of symptomatic individuals are assumed to need hospital treatment, with a distributed lag between infection and hospitalisation.
2. **ICU Admissions:** Similarly, an age-dependent fraction of symptomatic individuals are assumed to need treatment in an Intensive Care Unit. This is not a quantity that is generally reported, and therefore we cannot match our model predictions to this data source.
3. **Hospital Beds Occupied:** By convolving hospital admissions with the distributions of lengths of stay, we can estimate the number of hospital beds occupied.
4. **ICU Beds Occupied:** A similar process generates the number of occupied ICU beds.
5. **Number of Deaths:** Mortality is assumed to occur to a fraction of hospitalised individuals, with the probability of mortality dependent upon age, and occuring after a distributed lag.
6. **Proportion of Pillar 2 positives:** Given that the raw number of detected cases in any region is substantially influenced by the number of tests conducted, we consider the proportion of pillar 2 tests that are positive as a less biased figure. We assume that those symptomatically infected with COVID-19 compete with individuals suffering symptoms for other infections for the available testing capacity. This leads to proportion of pillar 2 tests that are positive being a saturating function of the number of symptomatic infections, with a single scaling parameter.

We compared these model predictions to the data by assuming that the true numbers are drawn from a negative binomial distribution with the model value as the mean, while the true proportions (Pillar 2 positives) are from a beta-binomial.

3 Parameter Inference

As with any model of this complexity, there are multiple parameters that determine the dynamics. Some of these are global parameters and apply for all geographical regions, with others used to capture the regional dynamics. Some of these parameters are matched to the early outbreak data (including the resultant age-distribution of infection), however the majority are inferred by an MCMC process (Table 1).

Table A: Key model parameters and their source

Parameter	Description	Source
β	Age-dependent transmission, split into household, school, work and other	Matrices from Prem *et al.* [4]
γ	Recovery rate, changes with τ, the relative level of transmission from undetected asymptomatics compared to detected symptomatics	Fitted from early age-stratified UK case data to match growth rate and R_0
$d_{a,v2}$	Age-dependent and type 2 vaccine status dependent probability of displaying symptoms (and hence being detected), changes with α and τ	Fitted from early age-stratified UK case data to capture the age profile of infection.
$\sigma_{a,v1}$	Age-dependent and type 1 vaccine status dependent susceptibility, changes with α and τ	Fitted from early age-stratified UK case data to capture the age profile of infection.
H^R	Household quarantine proportion $= 0.8\phi_R$	Can be varied according to scenario
N_a^R	Population size of a given age within each region	ONS
ε	Rate of progression to infectious disease ($1/\varepsilon$ is the duration in the exposed class). $\varepsilon \sim 0.2$	MCMC
α	Scales the degree to which age-structured heterogeneity is due to age-dependent probability of symptoms ($\alpha = 0$) or age-dependent susceptibility ($\alpha = 1$)	MCMC
τ	Relative level of transmission from asymptomatic compared to symptomatic infection	MCMC
ϕ^R	Regional relative strength of the lockdown restrictions; scales the transmission matrices. Can also be varied according to scenario.	MCMC
σ^R	Regional modifier of susceptibility to account for differences in level of social mixing	MCMC
E_0^R	Initial regional level of infection, rescaled from early age-distribution of cases	MCMC
D_S^R	Regional scaling for the mortality probability P_a(Death|Hospitalised))	MCMC
$H_{S,v3}^R$	Regional scaling for the hospitalisation probability P_a(Hospitalised|Symptomatic))	MCMC dependent on type 3 vaccine status
$I_{S,v3}^R$	Regional scaling for the ICU probability P_a(ICU|Symptomatics))	MCMC dependent on type 3 vaccine status

We would highlight that the parameters of α and τ are key in determining age-structured behaviour and are therefore essential in quantifying the role of school children in transmission [7]. We argue that a low τ and a low α are the only combination that are consistent with the growing body of data suggesting that levels of seroprevalence show only moderate variation across age-ranges [8], yet children are unlikely to display major symptoms, suggesting their role in transmission may be lower than for other respiratory infections [9, 10].

Throughout the current epidemic, there has been noticeable heterogeneity between the different regions

of England and between the devolved nations. In particular, London is observed to have a large proportion of early cases and a relatively steeper decline in the subsequent lock-down than the other regions and the devolved nations. In our model this heterogeneity is captured through three regional parameters (D^R_S, $H^R_{S,v3}$ and $I^R_{S,v3}$) which act on the heterogeneous population pyramid of each region to generate key observables. It is via this variable through which we affect a type 3 vaccine, acting to prevent severe disease outcome.

4 Modelling social distancing

Age-structured contact matrices for the United Kingdom were obtained from Prem et al. [4] and used to provide information on household transmission (β^H_{ba}, with the subscript ba corresponding to transmission from age group b towards age group a), school-based transmission (β^S_{ba}), work-place transmission (β^W_{ba}) and transmission in all other locations (β^O_{ba}). We assumed that the suite of social-distancing and lockdown measured acted in concert to reduce the work, school and other matrices while increasing the strength of household contacts.

We capture the impact of social-distancing by defining new transmission matrices (B_{ba}) that represent the potential transmission in the presence of extreme lockdown. In particular, we assume that:

$$B^S_{ba} = q^S \beta^S_{ba}, \qquad B^W_{ba} = q^W \beta^W_{ba}, \qquad B^O_{ba} = q^O \beta^O_{ba},$$

while household mixing B^H is increased by up to a quarter to account for the greater time spent at home. We take $q^S = 0.05$, $q^W = 0.2$ and $q^O = 0.05$ to approximate the reduction in attendance at school, attendance at workplaces and engagement with shopping and leisure activities during the lock-down, respectively.

For a given compliance level, ϕ, we generate new transmission matrices as follows:

$$\begin{aligned}
\widehat{\beta}^H_{ba} &= (1-\phi)\beta^H_{ba} + \phi B^H_{ba} \\
\widehat{\beta}^S_{ba} &= (1-\phi)\beta^S_{ba} + \phi B^S_{ba} \\
\widehat{\beta}^W_{ba} &= (1-\theta)\left[(1-\phi)\beta^W_{ba} + \phi B^W_{ba}\right] + \theta\left((1-\phi) + \phi q^W\right)((1-\phi) + \phi q^O)\beta^W_{ba} \\
\widehat{\beta}^O_{ba} &= \beta^O_{ba}((1-\phi) + \phi q^O)^2
\end{aligned}$$

As such, home and school interactions are scaled between their pre-lockdown values (β) and post-lockdown limits (B) by the scaling parameter ϕ. Work interactions that are not in public-facing 'industries' (a proportion 1 - θ) were also assumed to scale in this manner; while those that interact with the general populations (such as shop-workers) were assumed to scale as both a function of their reduction and the reduction of others. We have assumed $\theta = 0.3$ throughout. Similarly, the reduction in transmission in other settings (generally shopping and leisure) has been assumed to scale with the reduction in activity of both members of any interaction, giving rise to a squared term.

We infer the level of NPIs as a slowly varying parameter in the MCMC processes on a weekly basis. In turn, the weekly value of ϕ allows us to calculate the growth rate r (and hence the reproductive number R) by an eigenvalue approach.

5 QALY losses

Our computation of loss of quality adjusted life years (QALYs) incorporated loss due to deaths and losses associated with severe cases requiring hospitalisation.

QALY losses due to death were based on the quality adjusted life expectancy by age, modified for the relative life-expectancy of individuals that die:

$$\text{Fatal case QALY loss} = \sum_{a=1}^{21} \left(D(a) \times E(a)\right),$$

where $D(a)$ is the number of deaths in age bracket a, and $E(a)$ is the discounted quality adjusted value of the remaining life expectancy, $L(a)$, of individuals in age group a. This quality adjusted life expectancy is given by:

$$E(a) = \sum_{i=1}^{L(a)} \frac{Q_w(\hat{a}+i)}{(1+d)^i}$$

where $Q_w(a)$ is the age-specific quality of life weight at age a, $\hat{a}$ is the average age (in years) of an individual in age-group a, and d the discount rate (set at 0.035, corresponding to 3.5% per annum); the values of $L(a)$ are rounded to full years.

For individuals that are admitted to hospital (or ICU) we make the pessimistic assumption that their quality of life while in hospital is zero:

$$\text{Hospitalised QALY loss} = \sum_{a=1}^{21} \left(H(a) \times Q_w(a) \times \overline{H_S}\right),$$

where $H(a)$ is the number of hospital admissions in age bracket a, and $\overline{H_S}$ is the average hospital stay (approximately 10 days). We ignore the impact of recovery time outside the hospital and the effects of long-COVID. In all our calculations QALY loss from mortality vastly outweighs loss from hospital admissions.

For parameterising the age-specific quality of life weights, Q_w, we obtained age-specific EQ-5D index population norms estimates for England from two literature sources. We took childhood estimates (which we used to cover 0–19 years of age) from Table 3 of [11], and values for those aged 20 and above were sourced from Table 3.6 of [12]. A complete listing of age-specific quality of life weights values by age is presented in Table B.

Table B: EQ-5D index population norms for England.

Age group (yrs)	EQ-5D index population norms scale
<20	0.948
20–24	0.929
25–34	0.919
35–44	0.893
45–54	0.855
55–64	0.810
65–74	0.773
75+	0.703

Reference

1. Keeling MJ, Hill EM, Gorsich EE, Penman B, Guyver-Fletcher G, Holmes A, et al. Predictions of COVID-19 dynamics in the UK: Short-term forecasting and analysis of potential exit strategies. PLOS Comput Biol. 2021;17(1):e1008619. doi:10.1371/journal.pcbi.1008619.
2. Keeling MJ, Dyson L, Guyver-Fletcher G, Holmes A, Semple MG, Tildesley MJ, et al. Fitting to the UK COVID-19 outbreak, short-term forecasts and estimating the reproductive number. medRxiv. 2020; p. 2020.08.04.20163782. doi:10.1101/2020.08.04.20163782.
3. Mossong J, Hens N, Jit M, Beutels P, Auranen K, Mikolajczyk R, et al. Social Contacts and Mixing Patterns Relevant to the Spread of Infectious Diseases. PLoS Med. 2008;5(3):e74. doi:10.1371/journal.pmed.0050074.
4. Prem K, Cook AR, Jit M. Projecting social contact matrices in 152 countries using contact surveys and demographic data. PLOS Comput Biol. 2017;13(9):e1005697. doi:10.1371/journal.pcbi.1005697.
5. House T, Keeling MJ. Deterministic epidemic models with explicit household structure. Mathematical biosciences. 2008;213(1):29–39.
6. Hilton J, Keeling MJ. Incorporating household structure and demography into models of endemic disease. Journal of the Royal Society Interface. 2019;16(157):20190317.
7. Keeling MJ, Tildesley MJ, Atkins BD, Penman B, Southall E, Guyver-Fletcher G, et al. The impact of school reopening on the spread of COVID-19 in England. medRxiv. 2020; p. 2020.06.04.20121434. doi:10.1101/2020.06.04.20121434.
8. Pollán M, Pérez-Gómez B, Pastor-Barriuso R, Oteo J, Hernán MA, Pérez-Olmeda M, et al. Prevalence of SARS-CoV-2 in Spain (ENE-COVID): a nationwide, population-based seroepidemiological study. The Lancet. 2020;396(10250):535–544.
9. Fontanet A, Tondeur L, Madec Y, Grant R, Besombes C, Jolly N, et al. Cluster of COVID-19 in northern France: A retrospective closed cohort study. medRxiv. 2020; p. 2020.04.18.20071134. doi:10.1101/2020.04.18.20071134.
10. Heavey L, Casey G, Kelly C, Kelly D, McDarby G. No evidence of secondary transmission of COVID-19 from children attending school in Ireland, 2020. Eurosurveillance. 2020;25(21):pii=2000903. doi:10.2807/1560-7917.ES.2020.25.21.2000903.
11. Kwon J, Kim SW, Ungar WJ, Tsiplova K, Madan J, Petrou S. A Systematic Review and Meta-analysis of Childhood Health Utilities. Med Decis Mak. 2018;38(3):277–305. doi:10.1177/0272989X17732990.
12. Janssen B, Szende A. Population Norms for the EQ-5D. In: Self-Reported Popul. Heal. An Int. Perspect. based EQ-5D. Dordrecht: Springer Netherlands; 2014. p. 19–30.
